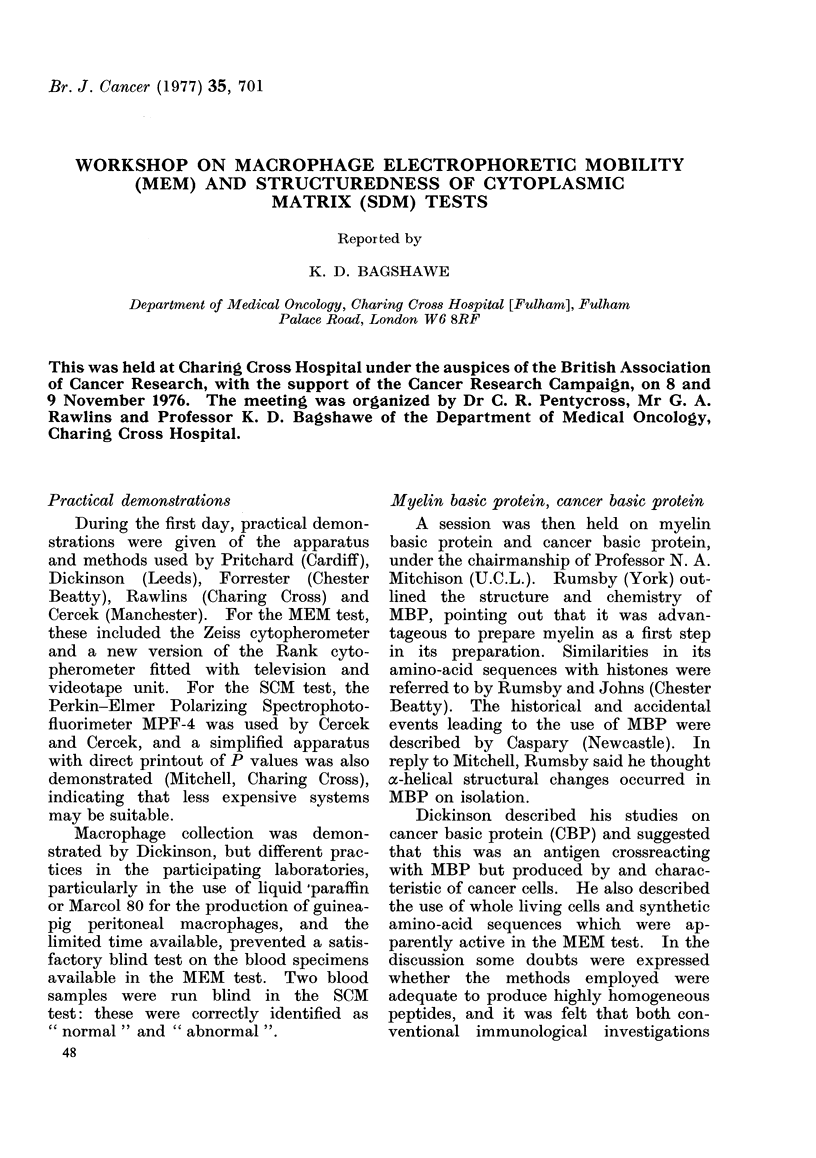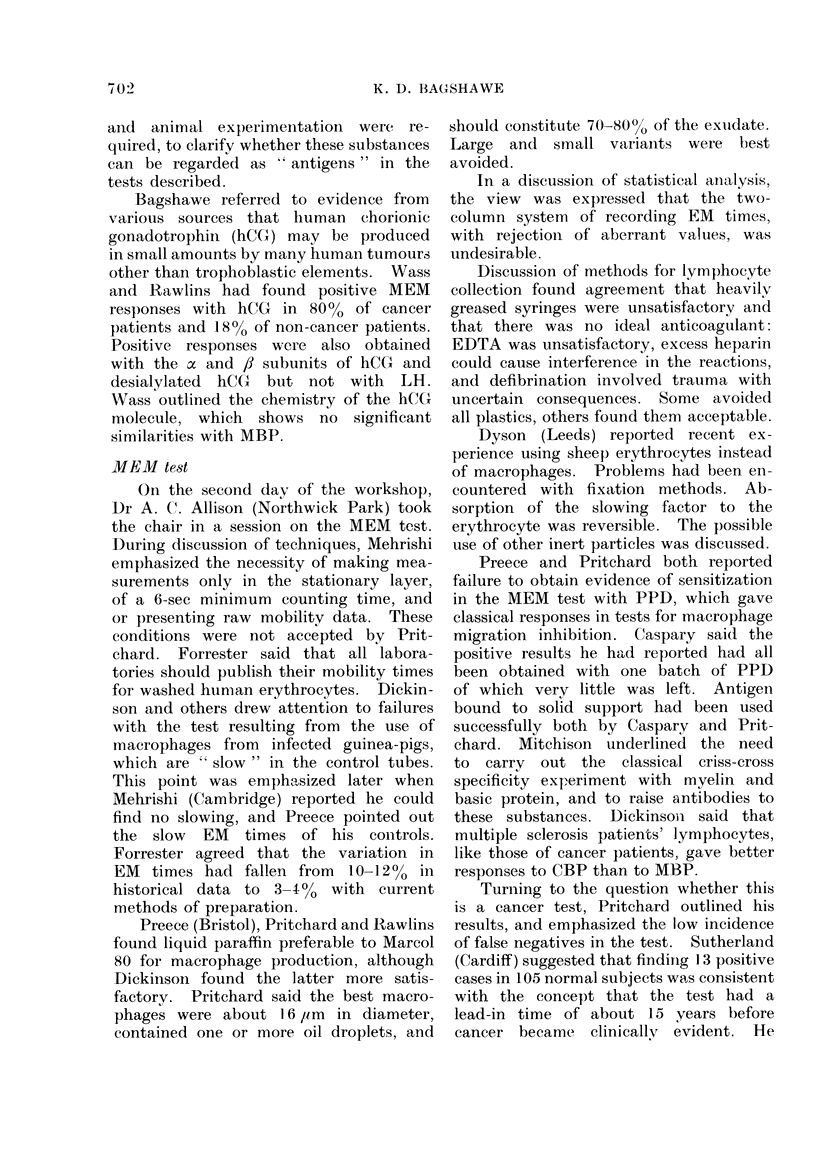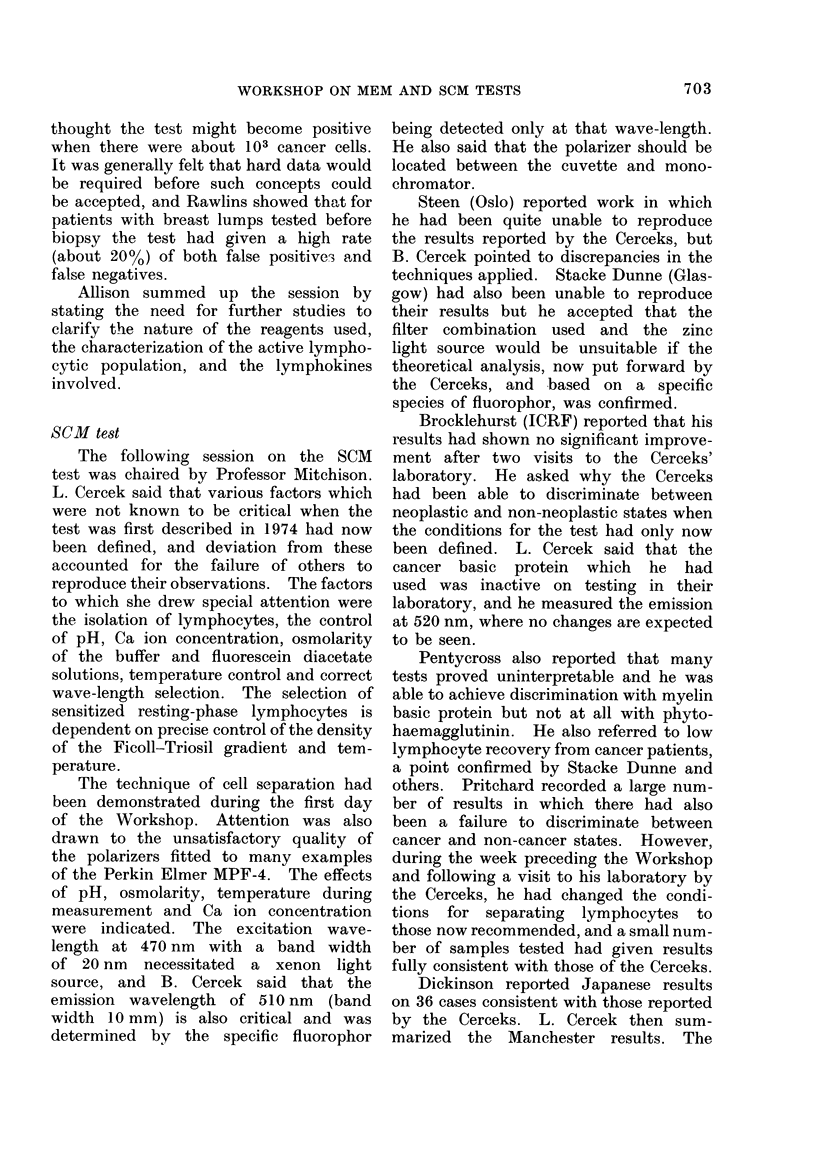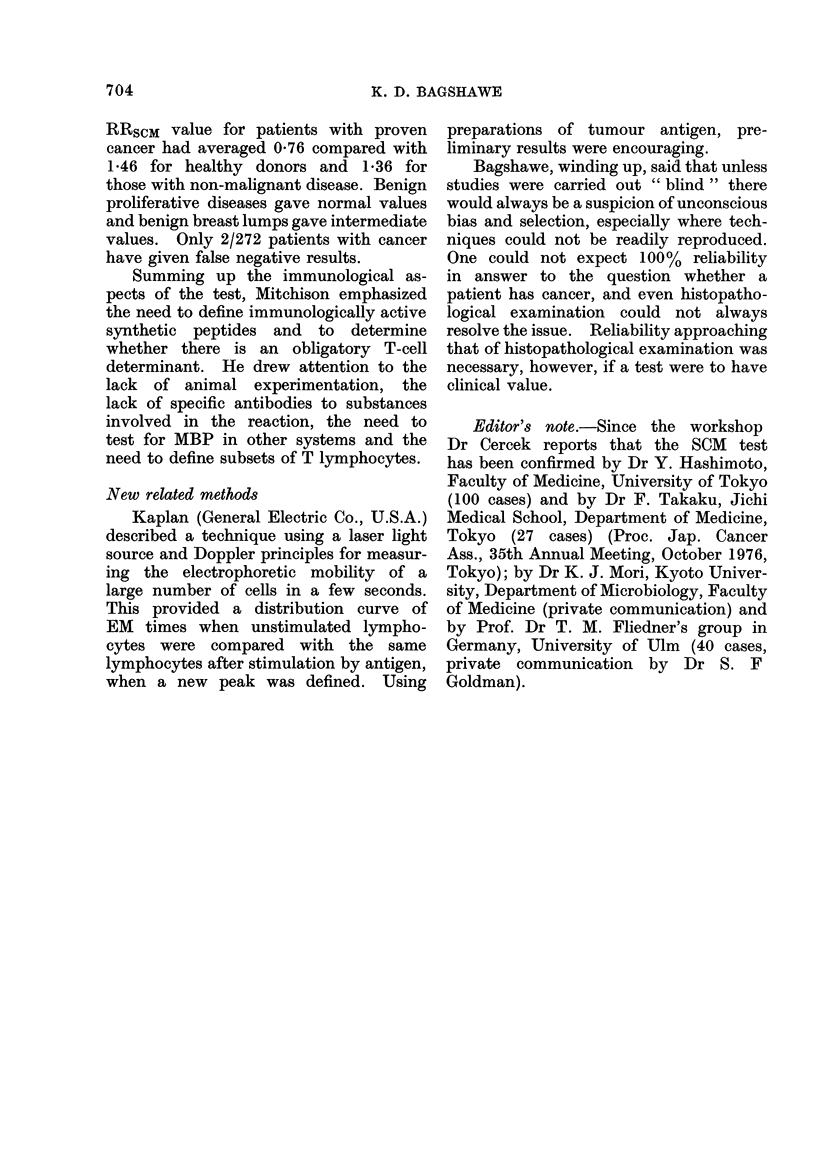# Workshop on Macrophage Electrophoretic Mobility (MEM) and Structuredness of Cytoplasmic Matrix (SDM) Tests

**Published:** 1977-05

**Authors:** 


*Br. J. Cancer* (1977) **35**, 701

### WORKSHOP ON MACROPHAGE ELECTROPHORETIC MOBILITY (MEM) AND STRUCTUREDNESS OF CYTOPLASMIC MATRIX (SDM) TESTS

Reported by

K. D. BAGSHAWE

*Department of Medical Oncology, Charing Cross Hospital [Fulham], Fulham Palace Road, London W6 8RF*

**This was held at Charing Cross Hospital under the auspices of the British Association of Cancer Research, with the support of the Cancer Research Campaign, on 8 and 9 November 1976. The meeting was organized by Dr C. R. Pentycross, Mr G. A. Rawlins and Professor K. D. Bagshawe of the Department of Medical Oncology, Charing Cross Hospital.**

*Practical demonstrations*

During the first day, practical demonstrations were given of the apparatus and methods used by Pritchard (Cardiff), Dickinson (Leeds), Forrester (Chester Beatty), Rawlins (Charing Cross) and Cercek (Manchester). For the MEM test, these included the Zeiss cytopherometer and a new version of the Rank cytopherometer fitted with television and videotape unit. For the SCM test, the Perkin–Elmer Polarizing Spectrophotofluorimeter MPF-4 was used by Cercek and Cercek, and a simplified apparatus with direct printout of *P* values was also demonstrated (Mitchell, Charing Cross), indicating that less expensive systems may be suitable.

Macrophage collection was demonstrated by Dickinson, but different practices in the participating laboratories, particularly in the use of liquid paraffin or Marcol 80 for the production of guinea-pig peritoneal macrophages, and the limited time available, prevented a satisfactory blind test on the blood specimens available in the MEM test. Two blood samples were run blind in the SCM test: these were correctly identified as " normal " and " abnormal ".

*Myelin basic protein, cancer basic protein*

A session was then held on myelin basic protein and cancer basic protein, under the chairmanship of Professor N. A. Mitchison (U.C.L.). Rumsby (York) outlined the structure and chemistry of MBP, pointing out that it was advantageous to prepare myelin as a first step in its preparation. Similarities in its amino-acid sequences with histones were referred to by Rumsby and Johns (Chester Beatty). The historical and accidental events leading to the use of MBP were described by Caspary (Newcastle). In reply to Mitchell, Rumsby said he thought $\alpha$-helical structural changes occurred in MBP on isolation.

Dickinson described his studies on cancer basic protein (CBP) and suggested that this was an antigen crossreacting with MBP but produced by and characteristic of cancer cells. He also described the use of whole living cells and synthetic amino-acid sequences which were apparently active in the MEM test. In the discussion some doubts were expressed whether the methods employed were adequate to produce highly homogeneous peptides, and it was felt that both conventional immunological investigations and animal experimentation were required, to clarify whether these substances can be regarded as "antigens" in the tests described.

Bagshawe referred to evidence from various sources that human chorionic gonadotrophin (hCG) may be produced in small amounts by many human tumours other than trophoblastic elements. Wass and Rawlins had found positive MEM responses with hCG in 80% of cancer patients and 18% of non-cancer patients. Positive responses were also obtained with the $\alpha$ and $\beta$ subunits of hCG and desialylated hCG but not with LH. Wass outlined the chemistry of the hCG molecule, which shows no significant similarities with MBP.

*MEM test*

On the second day of the workshop, Dr A. C. Allison (Northwick Park) took the chair in a session on the MEM test. During discussion of techniques, Mehrishi emphasized the necessity of making measurements only in the stationary layer, of a 6-sec minimum counting time, and or presenting raw mobility data. These conditions were not accepted by Pritchard. Forrester said that all laboratories should publish their mobility times for washed human erythrocytes. Dickinson and others drew attention to failures with the test resulting from the use of macrophages from infected guinea-pigs, which are "slow" in the control tubes. This point was emphasized later when Mehrishi (Cambridge) reported he could find no slowing, and Preece pointed out the slow EM times of his controls. Forrester agreed that the variation in EM times had fallen from 10–12% in historical data to 3–4% with current methods of preparation.

Preece (Bristol), Pritchard and Rawlins found liquid paraffin preferable to Marcol 80 for macrophage production, although Dickinson found the latter more satisfactory. Pritchard said the best macrophages were about 16 $\mu$m in diameter, contained one or more oil droplets, and should constitute 70–80% of the exudate. Large and small variants were best avoided.

In a discussion of statistical analysis, the view was expressed that the two-column system of recording EM times, with rejection of aberrant values, was undesirable.

Discussion of methods for lymphocyte collection found agreement that heavily greased syringes were unsatisfactory and that there was no ideal anticoagulant: EDTA was unsatisfactory, excess heparin could cause interference in the reactions, and defibrination involved trauma with uncertain consequences. Some avoided all plastics, others found them acceptable.

Dyson (Leeds) reported recent experience using sheep erythrocytes instead of macrophages. Problems had been encountered with fixation methods. Absorption of the slowing factor to the erythrocyte was reversible. The possible use of other inert particles was discussed.

Preece and Pritchard both reported failure to obtain evidence of sensitization in the MEM test with PPD, which gave classical responses in tests for macrophage migration inhibition. Caspary said the positive results he had reported had all been obtained with one batch of PPD of which very little was left. Antigen bound to solid support had been used successfully both by Caspary and Pritchard. Mitchison underlined the need to carry out the classical criss-cross specificity experiment with myelin and basic protein, and to raise antibodies to these substances. Dickinson said that multiple sclerosis patients' lymphocytes, like those of cancer patients, gave better responses to CBP than to MBP.

Turning to the question whether this is a cancer test, Pritchard outlined his results, and emphasized the low incidence of false negatives in the test. Sutherland (Cardiff) suggested that finding 13 positive cases in 105 normal subjects was consistent with the concept that the test had a lead-in time of about 15 years before cancer became clinically evident. He thought the test might become positive when there were about $10^3$ cancer cells. It was generally felt that hard data would be required before such concepts could be accepted, and Rawlins showed that for patients with breast lumps tested before biopsy the test had given a high rate (about 20%) of both false positives and false negatives.

Allison summed up the session by stating the need for further studies to clarify the nature of the reagents used, the characterization of the active lymphocytic population, and the lymphokines involved.

*SCM test*

The following session on the SCM test was chaired by Professor Mitchison. L. Cercek said that various factors which were not known to be critical when the test was first described in 1974 had now been defined, and deviation from these accounted for the failure of others to reproduce their observations. The factors to which she drew special attention were the isolation of lymphocytes, the control of pH, Ca ion concentration, osmolarity of the buffer and fluorescein diacetate solutions, temperature control and correct wave-length selection. The selection of sensitized resting-phase lymphocytes is dependent on precise control of the density of the Ficoll–Triosil gradient and temperature.

The technique of cell separation had been demonstrated during the first day of the Workshop. Attention was also drawn to the unsatisfactory quality of the polarizers fitted to many examples of the Perkin Elmer MPF-4. The effects of pH, osmolarity, temperature during measurement and Ca ion concentration were indicated. The excitation wavelength at 470 nm with a band width of 20 nm necessitated a xenon light source, and B. Cercek said that the emission wavelength of 510 nm (band width 10 mm) is also critical and was determined by the specific fluorophor being detected only at that wave-length. He also said that the polarizer should be located between the cuvette and monochromator.

Steen (Oslo) reported work in which he had been quite unable to reproduce the results reported by the Cerceks, but B. Cercek pointed to discrepancies in the techniques applied. Stacke Dunne (Glasgow) had also been unable to reproduce their results but he accepted that the filter combination used and the zinc light source would be unsuitable if the theoretical analysis, now put forward by the Cerceks, and based on a specific species of fluorophor, was confirmed.

Brocklehurst (ICRF) reported that his results had shown no significant improvement after two visits to the Cerceks' laboratory. He asked why the Cerceks had been able to discriminate between neoplastic and non-neoplastic states when the conditions for the test had only now been defined. L. Cercek said that the cancer basic protein which he had used was inactive on testing in their laboratory, and he measured the emission at 520 nm, where no changes are expected to be seen.

Pentycross also reported that many tests proved uninterpretable and he was able to achieve discrimination with myelin basic protein but not at all with phytohaemagglutinin. He also referred to low lymphocyte recovery from cancer patients, a point confirmed by Stacke Dunne and others. Pritchard recorded a large number of results in which there had also been a failure to discriminate between cancer and non-cancer states. However, during the week preceding the Workshop and following a visit to his laboratory by the Cerceks, he had changed the conditions for separating lymphocytes to those now recommended, and a small number of samples tested had given results fully consistent with those of the Cerceks.

Dickinson reported Japanese results on 36 cases consistent with those reported by the Cerceks. L. Cercek then summarized the Manchester results. The

$RR_{SCM}$ value for patients with proven cancer had averaged 0·76 compared with 1·46 for healthy donors and 1·36 for those with non-malignant disease. Benign proliferative diseases gave normal values and benign breast lumps gave intermediate values. Only 2/272 patients with cancer have given false negative results.

Summing up the immunological aspects of the test, Mitchison emphasized the need to define immunologically active synthetic peptides and to determine whether there is an obligatory T-cell determinant. He drew attention to the lack of animal experimentation, the lack of specific antibodies to substances involved in the reaction, the need to test for MBP in other systems and the need to define subsets of T lymphocytes.

*New related methods*

Kaplan (General Electric Co., U.S.A.) described a technique using a laser light source and Doppler principles for measuring the electrophoretic mobility of a large number of cells in a few seconds. This provided a distribution curve of EM times when unstimulated lymphocytes were compared with the same lymphocytes after stimulation by antigen, when a new peak was defined. Using preparations of tumour antigen, preliminary results were encouraging.

Bagshawe, winding up, said that unless studies were carried out " blind " there would always be a suspicion of unconscious bias and selection, especially where techniques could not be readily reproduced. One could not expect 100% reliability in answer to the question whether a patient has cancer, and even histopathological examination could not always resolve the issue. Reliability approaching that of histopathological examination was necessary, however, if a test were to have clinical value.

*Editor's note.*—Since the workshop Dr Cercek reports that the SCM test has been confirmed by Dr Y. Hashimoto, Faculty of Medicine, University of Tokyo (100 cases) and by Dr F. Takaku, Jichi Medical School, Department of Medicine, Tokyo (27 cases) (Proc. Jap. Cancer Ass., 35th Annual Meeting, October 1976, Tokyo); by Dr K. J. Mori, Kyoto University, Department of Microbiology, Faculty of Medicine (private communication) and by Prof. Dr T. M. Fliedner's group in Germany, University of Ulm (40 cases, private communication by Dr S. F Goldman).